# ASSOCIATIONS BETWEEN 10-YEAR PHYSICAL FUNCTION TRAJECTORIES AND 24-HOUR ACTIVITY CYCLE BEHAVIORS IN OLDER ADULTS

**DOI:** 10.1093/geroni/igae098.3804

**Published:** 2024-12-31

**Authors:** Mikael Anne Greenwood-Hickman, Weiwei Zhu, Abisola Idu, Laura Harrington, Susan McCurry, Andrea LaCroix, Pamela Shaw, Dori Rosenberg

**Affiliations:** Kaiser Permanente Washington Health Research Institute, Seattle, Washington, United States; Kaiser Permanente Washington Health Research Institute, Seattle, Washington, United States; Kaiser Permanente Washington Health Research Institute, Seattle, Washington, United States; Kaiser Permanente Washington Health Research Institute, Seattle, Washington, United States; University of Washington, Seattle, Washington, United States; University of California, San Diego, San Diego, California, United States; Kaiser Permanente Washington Health Research Institute, Seattle, Washington, United States; Kaiser Permanente Washington Health Research Institute, Seattle, Washington, United States

## Abstract

Physical activity (PA), sedentary behavior (SB), and sleep comprise an individual’s 24-hour activity cycle, which is likely bidirectionally associated with physical function over the life course. To explore how function predicts activity, we examined whether self-reported (activities of daily living [ADLs]) and objective (short Performance-based Physical Function [sPPF]) measures of physical function predicted PA, SB, and sleep in the Adult Changes in Thought (ACT) cohort. We fit linear mixed effects models to define trajectory slopes for each functional measure over the prior 10 years. We used multivariable linear regression to investigate the relationship between trajectories and device-measured PA and SB and self-reported sleep. Participants (N=905) were 77.6 (SD=6.9) years old, 55% female, 91% White, and reported impairment on a median of 1 ADL (IQR=[0,2]) with a median score of 9 (IQR=[8,11], higher=better functioning) on the sPPF at activity measurement. A steeper increasing slope of ADL impairment was associated with more sitting time (35.0 mins/day [4.3,65.0] per 0.4-unit increase in slope), longer mean sitting bout duration (3.5 min/bout [0.8,6.2]), fewer steps (-1372 [-2223,-638]), less MVPA (-13 min/day [-22.6,-5.0]), and more time in bed (25.5 min/day [6.5,43.5]). More steeply decreasing sPPF slope was associated only with steps and MVPA (-1180 steps [-2853,-185] and -15.7 min/day [-35.6,-2.3] per 0.3-unit decrease in slope). Standing time, Light-intensity PA, and self-reported sleep quality were not associated with either measure. Combined with prior literature suggesting associations with future function, these findings support the bidirectional nature of the relationship between the late-life 24-hour activity cycle and physical function.

